# Commentary: Characteristics and Long-Term Ablation Outcomes of Supraventricular Arrhythmias in Hypertrophic Cardiomyopathy: A 10-Year, Single-Center Experience

**DOI:** 10.3389/fcvm.2022.831343

**Published:** 2022-03-15

**Authors:** Rui-Huan Shen, Tong Zou

**Affiliations:** Department of Cardiovascular Medicine, National Center of Gerontology and Beijing Hospital, Chinese Academy of Medical Sciences, Peking Union Medical College, Beijing, China

**Keywords:** atrial fibrillation, hypertrophic cardiomyopathy, random error, survival analysis, Kaplan-Meier

## Introduction

This paper is a commentary on the article “Characteristics and Long-Term Ablation Outcomes of Supraventricular Arrhythmias in Hypertrophic Cardiomyopathy: A 10-Year, Single-Center Experience (1).” The authors aimed to identify a cut-off value of left ventricular end-diastolic dimension (LVEDD) to predict atrial fibrillation (AF) recurrence after catheter ablation in patients with hypertrophic cardiomyopathy (HCM). However, there are inconsistencies between Figures 3A,B in numbers at risk, as well as, between the Kaplan-Meier curve and corresponding numbers at risk in the Figure 3B in the original manuscript. Furthermore, the receiver operative characteristic curves (ROC) analysis to find an optimal cut-off is not a reliable or accurate approach with such a small sample size (*n* = 16). Therefore, the conclusion of Zhang et al. that a greater LVEDD predicts AF recurrence after catheter ablation in patients with HCM may be unreliable.

## Main Text

In the original manuscript, the inconsistency in numbers at risk between Figures 3A,B, as well as the inconsistency between the Kaplan-Meier curve and corresponding numbers at risk in Figure 3B, ought to get our attention. Firstly, in Figure 3A there are 15 patients at 12 months of follow-up in the Kaplan-Meier curve, while in Figure 3B, there are only 8 patients. Among them are 4 patients in the subgroup with LVEDD <47 mm and 4 patients with LVEDD ≥ 4.7 mm. Hence, there are 7 patients missing in Figure 3B at the 12-month time point.

As a matter of fact, there are further inconsistencies: in Figure 3A, a proportion of 1/3 among the 15 patients remaining at the 24-month time point has had a relapse, i.e., 5 patients, and according to the text, the subgroup with LVEDD <47 mm does not appear to have experienced any events during the follow-up period, so all of those had LVEDD ≥ 47 mm. However, in Figure 3B, there were only 7 patients fulfilling the criterion of LVEDD ≥ 47 mm, and of those, 4 are remaining in the curve at the 12-month time point suggesting only 3 patients with LVEDD ≥ 47 mm had relapsed into AF at that point.

Secondly, as described in the original manuscript, Figure 3B shows that “In Kaplan-Meier analysis, patients with a LVEDD ≥ 47 mm had a worse AF-free survival than those with a LVEDD <47 mm (log-rank *p* = 0.014) (1)”. As the Figure 3B shows that of the 16 patients who had AF ablation, although, the cumulative AF-free survival in the subgroup with LVEDD <47 mm is a straight line, which means that the subgroup does not appear to have experienced any events during the follow-up period, the corresponding Number at risk, at the bottom of the Figure 3B, in LVEDD <47 mm subgroup does not match to the straight line. Moreover, it is difficult to believe that log-rank testing would be significant with such small numbers in the Kaplan-Meier curve.

Only 19% of their ablation patients have two or more years of follow-up. Hence, the title is misleading as there is not really a meaningful 10-year follow-up to show.

Furthermore, to determine an optimal cut-off value of LVEDD by receiver operating characteristic (ROC) analysis to predict AF recurrence after catheter ablation in patients with HCM in a sample size of 16 persons may be a kind of irrationality, which causes the occurrence of random error. In consequence, the conclusion of Zhang et al. that a greater LVEDD predicts AF recurrence after catheter ablation in patients with HCM may be unreliable. Our task group hopes the authors can give a reasonable explanation.

## Discussion

Keeping the data consistent with the figure helps the reader not to be confused and is more conducive to the exchange of the author's clinical experience. When ROC was used to determine the optimal cut-off values of predictors, a larger sample size may make the conclusion more reliable.

## Author Contributions

All authors listed have made a substantial, direct, and intellectual contribution to the work and approved it for publication.

## Funding

This work was supported by Beijing Municipal Science & Technology Commission Program (Nos. Z171100001017203 and D181100000218005).

## Conflict of Interest

The authors declare that the research was conducted in the absence of any commercial or financial relationships that could be construed as a potential conflict of interest.

## Publisher's Note

All claims expressed in this article are solely those of the authors and do not necessarily represent those of their affiliated organizations, or those of the publisher, the editors and the reviewers. Any product that may be evaluated in this article, or claim that may be made by its manufacturer, is not guaranteed or endorsed by the publisher.
